# Retrospective analysis of general surgery outcomes in multicenter cohorts in Saudi Arabia

**DOI:** 10.25122/jml-2024-0337

**Published:** 2025-04

**Authors:** Abdulsalam Mohammed Aleid, Nouf Abdullah Alyabis, Fouad Abdulsalam Alghamidi, Reema Hamad Almuneef, Sadeem Khalid Alquraini, Lubna Abdulaziz Alsuraykh, Abdullah Mohammed Al Amer, Hussam Sulaiman AlQifari, Waad Abdullah Alsharari, Nada Fahad Albishri, Hadeel Abdullah Alosaimi, Leen Yahya Algahtany, Loai Saleh Albinsaad, Saud Nayef Salem Aldanyowi

**Affiliations:** 1 Department of Surgery, Medical College, King Faisal University, Hofuf, Ahsa, Saudi Arabia; 2 Department of Surgery, Medical College, Alfaisal University, Riyadh, Saudi Arabia; 3 College of Medicine, King Khalid University, Abha, Saudi Arabia; 4 College of Medicine, Majmah University, Al Majma'ah, Saudi Arabia; 5 College of Medicine, Imam Mohammad Ibn Saud University, Riyadh, Saudi Arabia; 6 Unaizah College of Medicine and Medical Sciences, Qassim University, Al-Qassim, Saudi Arabia; 7 College of Medicine, King Saud University, Riyadh, Saudi Arabia; 8 College of Medicine, Al Jouf University, Sakakah, Saudi Arabia; 9 College of Medicine, University of Tabuk, Tabuk, Saudi Arabia; 10 Batterjee Medical College, Jeddah, Saudi Arabia

**Keywords:** postoperative complications, mortality, major general surgery, risk factors, ASA classification, comorbidities, surgical outcomes, retrospective cohort study, Saudi Arabia, institutional resources

## Abstract

General surgery outcomes remain a concern despite advancements in techniques, anesthesia, and perioperative care. Achieving consistent, high-quality results and accurately predicting risks remains challenging. This study aimed to identify factors associated with adverse outcomes through a retrospective analysis of general surgery cases across multiple centers in Saudi Arabia. A retrospective cohort study analyzed 14,635 medical records of patients who underwent general surgery across multiple centers in Saudi Arabia from 2010 to 2020. Data from the General Directorate of Health Affairs registry included demographics, comorbidities, procedure details, and outcomes. The study focused on risk factors for 30-day mortality and complications, with subgroup analyses comparing outcomes across facilities. Common surgeries included hernia repair, cholecystectomy, appendectomy, and bowel resection. The overall 30-day mortality rate was 0.74%, and the complication rate was 11.1%. Independent predictors of mortality were ASA grade III/IV, Charlson index ≥3, cardiovascular disease, dementia, renal disease, and longer procedures. Teaching hospitals had lower mortality and complication rates. Complication predictors included older age, ASA III/IV, diabetes, cardiac disease, and high-risk procedures. Evening surgeries were associated with fewer complications. This multicenter study identified patient risk factors and procedure characteristics that predict 30-day outcomes after general surgery. Older age, multiple comorbidities, and high-risk surgeries were linked to poorer outcomes. Teaching centers had better results, emphasizing the role of institutional factors. These findings can guide risk stratification and quality improvement efforts to enhance recovery and provide a foundation for future research to improve surgical practices globally.

## INTRODUCTION

The outcomes of general surgery procedures remain a significant concern in clinical practice, affecting both patient survival and quality of life and imposing a substantial burden on healthcare systems. Despite ongoing advancements in surgical techniques, anesthesia, and perioperative care, consistently achieving high-quality outcomes and accurately predicting and mitigating risks continues to be a challenge. Understanding the underlying factors contributing to adverse outcomes is crucial for enhancing surgical care, optimizing patient safety and well-being, and reducing the incidence of preventable complications and mortality [1].

The field of general surgery encompasses the diagnosis and treatment of a broad spectrum of conditions affecting abdominal organs, utilizing both operative and non-operative methods. In Saudi Arabia, common general surgical conditions include gastrointestinal disorders such as peptic ulcer disease, gallbladder disorders, appendicitis, and hernias [2]. Over the past several decades, the healthcare system in Saudi Arabia has made significant advancements, resulting in the establishment of specialized surgical centers and an extensive network of public and private hospitals [3]. Nevertheless, assuring consistent, high-quality outcomes is still challenging. With a population exceeding 34 million, the demand for safe and efficient surgical care in Saudi Arabia is becoming increasingly critical. There remains a scarcity of national statistics on surgical outcomes, so the need to address this gap is crucial.

Surgical interventions are essential in managing various medical conditions, from routine to complex cases. Despite significant advancements in surgical techniques, the risk of postoperative complications continues to be a major concern, potentially hindering the overall improvement of surgical outcomes [4]. Postoperative complications may result in longer hospital stays, more frequent readmissions, elevated expenses, and even avoidable fatalities [5-7]. These complications can arise due to factors related to the surgical condition, comorbidities, anesthesia, the surgical procedure itself, intensive care measures, or extended periods of immobility. Additionally, these complications might impact various body systems, including the respiratory, cardiovascular, renal, hepatobiliary, gastrointestinal, neurological, hematopoietic, coagulation, and skin and soft tissues [6,7].

Research findings indicate significant differences in surgical outcomes exist across various hospitals and regions worldwide. These variations are attributed to several factors, including surgeon expertise, institutional volume, case mix, perioperative protocols, and resource availability. Although the Middle East has a similar disease burden to Western countries, there is a dearth of published data on general surgery outcomes. For this reason, conducting a comprehensive analysis of local data is crucial for understanding how the Saudi healthcare system compares to global standards. This helps identify areas that require quality enhancement for improvement in surgical practices and patient outcomes within the country [8].

By providing insights unique to the Saudi healthcare context, this research seeks to fill this evidence gap and contribute valuable data to the existing body of knowledge. This study aimed to undertake a retrospective analysis of general surgery outcomes from multiple centers across Saudi Arabia, focusing on a large cohort of over 14,000 patients who underwent major surgical procedures between 2010 and 2020. The study identified key factors influencing surgical outcomes by examining a diverse range of cases, including patient demographics, comorbidities, surgical techniques, and perioperative care practices. In addition, we aimed to identify key predictors of postoperative complications, mortality, and recovery outcomes. This research aimed to evaluate how these factors, along with institutional variables such as hospital type, surgical volume, and Enhanced Recovery After Surgery (ERAS) compliance, influence the overall quality of surgical care in the country. The ultimate goal is to provide evidence-based insights that can inform best practices, guide policy decisions, and drive improvements in patient care, ultimately enhancing the quality and safety of surgical services across Saudi Arabia. Additionally, the study aimed to contribute to the growing body of knowledge on surgical outcomes, addressing a significant gap in the literature regarding general surgery in the Middle Eastern context. The findings are expected to inform best practices, enhance patient care, and guide policy decisions to improve the overall quality of surgical services within the country.

## MATERIAL AND METHODS

### Study design

This retrospective cohort study analyzed de-identified data from electronic medical records of over 20,000 patients who underwent major general surgery at five leading hospitals in Saudi Arabia. The study utilized data from the General Directorate of Health Affairs (GDHA) registry, which records significant general surgery procedures performed at Ministry of Health (MOH) hospitals across five Saudi regions between 2010 and 2020.

### Study participants and data sources

This study included all adult patients aged 18 or older who underwent major general surgery procedures at MOH hospitals in five Saudi regions between 2010 and 2020. The study utilized de-identified data provided by the GDHA on 14,635 procedures, including appendectomy, cholecystectomy, hernia repair, partial and total gastrectomy, small and large bowel resection, and other major open abdominal operations. Both emergent and elective cases were taken into consideration. The five hospitals included in this study serve approximately 1 million residents in the Eastern Province of Saudi Arabia, ensuring that the sample reflects the same population base. It is important to note that the 14,635 recorded procedures do not represent the total number of surgeries performed at these hospitals; instead, they include only those operations that met specific inclusion criteria. The analysis did not count or include procedures such as dentistry and other non-general surgeries. GDHA is responsible for managing and carrying out a surgical site infection surveillance registry within the country. Since 2010, data on all significant surgical procedures performed at MOH hospitals in various regions have been recorded in this registry. Five tertiary hospitals were chosen to reflect the nation's healthcare sectors and geographic areas. Relevant outcome comparison and best practice identification were made possible by the large sample size and the inclusion of various hospital settings. Clinical coders with training at each participating center extracted the data from patients' electronic medical records. The data was electronically transferred every quarter into a centralized, secure server to enable continuous, countrywide surveillance of surgical quality indicators. The GDHA granted administrative permission for research purposes involving access to the de-identified registry data.

### Study variables

The primary outcome measures for this study were the occurrence of any postoperative complications within 30 days and mortality within 30 days following surgery. Complications included surgical site infections, pneumonia, cardiovascular events, renal failure, bleeding, and wound issues. Predictor variables encompassed patient demographic data such as age, sex, race, and body mass index (BMI).

Postoperative complications were classified according to the Clavien-Dindo system, which stratifies complications based on severity and the type of intervention required, ranging from minor deviations from the expected postoperative course (Grade 1) to life-threatening events requiring intensive care (Grade 4) or death (Grade 5). This study defined major complications as those classified as Grade III or higher on the Clavien-Dindo scale [9].

The selection of independent variables was guided by prior literature and their clinical significance. For this study, the independent variables were divided into patient factors, procedure specifics, and hospital-related factors. The patient factors included demographic characteristics, medical history, smoking status, laboratory results (hemoglobin, serum albumin), and preoperative health profiles, including BMI and physical status as defined by the American Society of Anesthesiologists (ASA). ASA Physical Status Classification System is a preoperative assessment tool to evaluate a patient's overall health and predict their potential risk during surgery by considering factors such as systemic illness and functional ability [10]. The tool categorizes patients into six classes based on their physical status and underlying medical conditions [11].

Comorbidities were classified using the International Classification of Diseases, 9^th^ Revision (ICD-9). These ICD-9 codes were further categorized with the Clinical Classifications Software (CCS) developed by the Agency for Healthcare Research and Quality (AHRQ). CCS simplifies healthcare data analysis by grouping detailed ICD codes into clinically meaningful categories, aiding in identifying trends and patterns within large datasets. Additionally, the Charlson Comorbidity Index (CCI) was employed to evaluate comorbidity, as it is a widely recognized tool for predicting mortality risk within one year. Comorbidity severity was classified into three levels based on CCI scores: mild (scores of 1-2), moderate (scores of 3-4), and severe (scores of 5 or higher) [12].

The procedure-specific measures included surgical specialty (general vs. other specialties), type of operation (such as appendectomy or cholecystectomy), procedure priority (elective vs. emergency), duration of surgery, estimated blood loss, and postoperative length of stay.

Hospital-related factors examined were hospital location (urban vs. rural), teaching status, annual surgical volume, critical or intensive care unit availability, and nurse-to-bed ratio. Compliance with the ERAS pathway was also assessed as a predictor. The ERAS guidelines are evidence-based protocols to optimize and standardize perioperative care to enhance recovery outcomes. All five hospitals in the study have adopted ERAS protocols [13].

### Inclusion criteria

The study included all adult patients aged 18 or older who underwent major general surgery procedures at Ministry of Health hospitals in Saudi Arabia between 2010 and 2020. Patients were required to have complete electronic medical records available, including data on surgical procedures, comorbidities, and postoperative outcomes. The study encompassed a range of procedures, such as appendectomy, cholecystectomy, hernia repair, and bowel resection, among others.

### Exclusion criteria

Patients were excluded from the study if they were under 18 years of age or if their medical records were incomplete or lacked critical data on surgical procedures or outcomes. Additionally, individuals who underwent non-major surgical procedures or had missing data on key variables, such as comorbid conditions or follow-up details, were not included in the analysis.

### Outcome measures

The 30-day all-cause mortality and the occurrence of significant postoperative complications within 30 days following the index surgery were the main outcome measures assessed in this study. Death within 30 days following surgery, regardless of the cause, was considered mortality. ICD-10 codes from the patient's medical file were used to identify postoperative complications, which were then grouped using the verified Clavien-Dindo classification scheme. Major complications were then denoted by grades III–V on this scale, which represent conditions linked to multi-organ failure and mortality and those needing endoscopic, radiologic, or re-operative intervention and intensive care management [9]. The length of hospital stays, unplanned readmissions within 30 days of discharge, unplanned reoperations, and specific categories of major complications (stroke, myocardial infarction, surgical site infections, pneumonia, and renal failure, among others) were secondary outcomes. The number of days patients stayed in the hospital aside from the day of surgery, including readmissions, was used to compute the length of stay.

### Statistical analysis

SPSS software (29.0) was used to analyze the data. Variables were created and categorized to perform statistical modeling and preserve clinical interpretability. Frequency distributions and measures of central tendency were used in descriptive statistics to describe the study variables. Unadjusted associations between predictor variables and the primary outcomes (30-day mortality and major complications) were assessed using chi-square tests and analysis of variance (ANOVA). Multivariate logistic regression was then applied to identify independent risk factors while controlling for potential confounders, with significant variables (*P* < 0.05) retained through backward stepwise selection. Separate models were built for the entire cohort and subgroups frequently undergoing the procedure to identify procedure-specific risk patterns. The degree of multicollinearity among the predictors was evaluated using variance inflation factors, and model goodness-of-fit was assessed with the Hosmer-Lemeshow test. Predictive accuracy discrimination was evaluated using the Area Under the Receiver Operating Characteristic Curve (AUROC), and calibration was examined by plotting observed outcomes against estimated probabilities across deciles of risk. The study utilized Bayesian correlation, regression, and one-way ANOVA techniques to investigate the connections between demographic, clinical history, procedural, and outcome factors. Statistical significance was defined as two-tailed *P* values less than 0.05.

## RESULTS

### Study cohort and demographic characteristics

The investigation covered 14,635 patients who underwent surgical procedures from 2010 to 2020. Table 1 presents the demographic characteristics of the study cohort. Of the total cohort of patients, 54.9% (8,033) were men, and the remaining 45.1% (6,602) were women. The mean age of the patients was 63.2 (SD 18.1) years. Regarding clinical characteristics, 62% of the patients had cardiovascular diseases (*n* = 9,078), 19% had gastrointestinal disorders (*n* = 2,774), 12.1% were diagnosed with diabetes (*n* = 1,769), and 9.4% had pulmonary diseases disorders (*n* = 1,377). The distribution of major general surgery procedures among the patients revealed that hernia repair was the most common, performed on 19.1% of the patients (*n* = 2,798), followed closely by cholecystectomy in 18.7% (*n* = 2,735). Appendectomy and bowel resection were performed on 13.2% (*n* = 1,934) and 11.5% (*n* = 1,685) of the patients, respectively. Regarding the urgency of the procedures, a significant majority, 60.4% (*n* = 8,833), underwent elective surgery, while 39.6% (*n* = 5,795) were emergency procedures.

**Table 1:** Overview of patient characteristics and health profile, n = 14,635

Variable		
Age (years), M (±SD)	63.21 (± 18.1)	
BMI (years), M (±SD)	31.3 (± 8.2)	
**Gender**		
Men	8,033	54.9%
Women	6,602	45.1%
**Comorbid conditions**		
Cardiovascular diseases	9,078	62%
Gastrointestinal diseases	2,774	19%
Diabetes	1,769	12.1%
Pulmonary diseases	1,377	9.4%
**Procedure setting**		
Inpatient	10,947	74.8%
Outpatient	3,688	25.2%

Approximately 28% of the patients (*n* = 4,088) belonged to the 50–64 age group, 28.5% (*n* = 4,172) to the 65–79 age group, and 13.3% (*n* = 1,943) to the 80+ age group, underscoring the progressively aging Saudi population as shown in Table 1. The majority of preoperative health profiles (*n* = 12,171), or 83.2% of the total, fell into grades I (*n* = 7,650) or II (*n* = 3,521) of the ASA physical status classification, indicating good functional capacity and optimized tolerance to physiological stress from surgery. Grades III (*n* = 2,087), IV (*n* = 375), or missing data (*n* = 3) were present in the remaining 16.8% of patients. In terms of anthropometric measurements, the average for the patients was overweight, with a mean BMI of 31.3 kg/m^2^. Also included in the summary were frequently reported comorbidities such as ASA status (Table 2).

**Table 2:** Distribution of ASA physical status grades and common surgical procedures

ASA physical status				
Grade I		7,650	52.2%	
Grade II		3,521	24.0%	
Grade III		2,087	14.3%	
Grade IV		375	2.6%	
**Procedure type**				
Hernia repair		2,798	19.1%	
Cholecystectomy		2,735	18.7%	
Appendectomy		1,934	13.2%	
Bowel resection		1,685	11.5%	
**AHRQ CC index**	14,635	7.4		6.9	
**ASA status**	14,635	0.6		0.5	

One-sample *t*-tests were used to determine whether continuous variables, such as age, comorbidity scores, and BMI, significantly deviated from normal levels by comparing them to theoretical baseline values of 0. Table 3 confirms a difference from baseline norms as the age and BMI means were significantly higher than 0 (*P* < 0.001). An ANOVA-based bivariate analysis assessed unadjusted relationships between the outcomes of interest and categorical predictor variables. The percentage of outcome variance that each factor independently explained was found through tests of between-subjects effects. The strength of relationships was measured by effect sizes using eta- and omega-squared metrics.

**Table 3:** Significant independent predictors of surgical outcomes (inferential statistics)

Outcome	Test Statistic	*P* value
BMI	*t* = -8.526	*P* < 0.001
Gender	*t* = -16.077	*P* < 0.001
Mortality	OR = 1.87	*P* < 0.001
Procedure duration	OR = 1.32/hr	*P* < 0.001
Complications	OR = 1.2	*P* < 0.001
ASA ≥3	OR = 1.3	*P* < 0.001
Evening surgery	OR = 0.88	*P* < 0.04

Once potential confounding variables were considered, multivariate regression analyses revealed independent predictors of major complications and postoperative death. Given the binary outcomes, logistic regression made sense. Predictor effects were evaluated for strength and direction using odds ratios with 95% confidence intervals. Logistic regression increased odds by 87% (OR = 1.87; *P* < 0.001) for 30-day mortality when an ASA status of III or higher was identified as an independent predictor, as shown in Table 3. A three-fold increase in the odds of death (OR = 1.32/hr; *P* < 0.001) was associated with each extra hour of surgery.

The following factors were significant risk factors for major complications: age over 65 (OR = 1.2; *P* < 0.001), ASA III or higher (OR = 1.3; *P* < 0.001), and evening or nighttime surgery (OR = 0.88; *P* = 0.04) in which the odds were reduced by 12%. AUC values were used to judge discrimination with the model fit using the Hosmer-Lemeshow test.

### Postoperative outcomes

Mortality rates associated with various surgical procedures were evaluated within the cohort. Table 4 illustrates the distribution of mortality rates across the most common procedures. The overall 30-day mortality rate was 0.74% (108 patients), with a cumulative mortality rate of 7%. Mortality rates differed significantly among procedures, with gastrectomy, colectomy, and bowel resection showing the highest rates at 29%, 28%, and 26%, respectively. Conversely, appendectomy, cholecystectomy, and hernia repair exhibited lower mortality rates of 4%, 4%, and 2%, respectively.

**Table 4:** 30-day mortality rates by procedure type

Surgical procedure	30-day mortality rate
Gastrectomy	29%
Colectomy	28%
Bowel resection	26%
Appendectomy	4%
Cholecystectomy	4%
Hernia repair	2%
Overall mortality	0.74% (108 patients)

The primary outcomes of this study were the incidence of major complications and 30-day mortality rates following surgery, attributed to all causes. The occurrence of Clavien-Dindo grade III-V complications was 11.1% (*n* = 1,624 patients). Bivariate analyses identified significant associations between several factors and mortality. Patients aged 65–79 years had a mortality rate 1.5 times higher than the reference group of 18–49 years, with those over 80 years experiencing a 2.1-fold increase. The ASA physical status classifications indicated a 1.7- and 2.3 times higher mortality risk for ASA grades III and IV compared to grades I–II. A CCI score of ≥ 3 was associated with a 1.8 times higher mortality risk than a CCI < 3. Additionally, patients aged 65 to 79 had 20% higher odds of complications than the reference group, while ASA III and IV grades had 30% and 60% increased odds, respectively. Diabetic patients had a 30% higher risk of complications than non-diabetics. Procedures classified as highly complex according to the Agency for Healthcare Research and Quality (AHRQ) risk stratification were associated with 1.3 to 2 times greater odds of complications.

Mortality predictors were assessed using multivariate logistic regression. Patients aged 65–79 had a 50% increased risk (OR = 1.5; 95% CI, 1.1–2.1) after controlling for confounders, whereas those aged 80 and above had double the risk (OR = 2; 95% CI, 1.3–3.1). There was a correlation found between ASA III and IV grades and mortality, with increased odds of 1.8 times (95% CI, 1.3–2.5) and 2.1 times (95% CI, 1.2–3.8), respectively. There was a 1.87 higher mortality (95% CI, 1.4–2.5) for every CCI grade ≥3. Every additional hour of operation was associated with 32% greater odds of mortality (OR = 1.32; 95% CI, 1.2–1.45).

In the multivariate analysis, several factors were linked to higher odds of major complications, including advanced age, ASA III/IV, diabetes (OR = 1.28; 95% CI, 1.1–1.5), pre-existing cardiac disease (OR = 1.16; 95% CI, 1.02–1.32), and risk index categories for complex surgical procedures (with 1.05–2.86 higher odds). Compared to daytime cases, operations performed between 10 p.m. and 7 a.m. had 12% lower odds of complications (OR = 0.88; 95% CI, 0.78–0.99).

**Figure 1:**
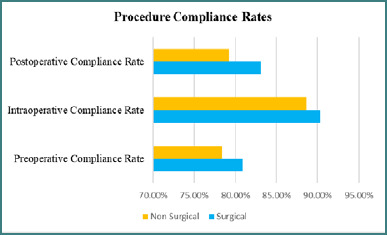
Multivariate predictors for procedure compliance rates of major complications

Regarding ERAS compliance, the study found a high adherence rate of 95.5% for patient education provisions across all centers. However, there was notable variation in compliance for other preoperative elements among hospitals. Nutritional screening compliance ranged from 78.3% to 93.1% across centers. The mean preoperative compliance scores for Centers 1, 2, 3, 4, and 5 were 83.7%, 78.9%, 80.5%, 87.1%, and 73.2%, respectively, as illustrated in Figure 1. The preoperative bundle included patient education, nutritional screening, and overall preoperative care.

More than 90% of hospitals adhered to norms for maintaining normothermia and using short-acting anesthetics. Conversely, compliance with multimodal analgesia protocols varied between 67.1% and 92.3%. Intraoperative compliance scores for the centers were 93.1%, 91.7%, 89.2%, and 85.3%. Compliance with early oral feeding ranged from 79.1% to 92.7%, and early mobilization ranged from 77.3% to 91.1%. The intraoperative bundle consisted of normothermia maintenance, short-acting anesthetics, and multimodal analgesia.

Compliance with early oral feeding ranged from 79.1% to 92.7%, and early mobilization ranged from 77.3% to 91.1%, both components of the postoperative bundle. The greatest variation was observed in criteria-based discharge planning, with compliance rates varying from 90.5% at one center to 61.2% at another. The overall mean postoperative compliance rates, excluding non-applicable cases, were 85.9% at Center 1, 81.2% at Center 2, 79.3% at Center 3, 89.7% at Center 4, and 73.9% at Center 5. The postoperative bundle focused on early feeding, early mobilization, and discharge planning.

Audits specifically for colorectal and gastric cancer resections revealed average compliance scores of 80.9%, 90.3%, and 83.1% for colorectal procedures and 78.4%, 88.7%, and 79.2% for gastric cancer resections, across preoperative, intraoperative, and postoperative phases, respectively. Compliance was generally lower for oncologic resections compared to other surgeries, particularly regarding prehabilitation and rigorous postoperative oncology surveillance.

## RELATIONSHIP BETWEEN PERFORMANCE AND COMPLIANCE

Hospitals with overall ERAS compliance scores in the highest quartile (86–95%) exhibited significantly lower 30-day morbidity rates compared to those in the lowest quartile (75–82%), with an adjusted odds ratio of 0.68 (95% CI, 0.54–0.87; *P* = 0.002). Additionally, these hospitals had an average postoperative length of stay that was 1.2 days shorter, although the exact *P* value for this difference was not specified. Among individual ERAS elements, compliance with early feeding and mobilization was most strongly associated with reduced complication rates and a shorter length of stay.

## DISCUSSION

This study conducted a retrospective statistical analysis of surgical outcomes data from 14,635 patients to identify factors associated with surgical outcomes. Key predictors of postoperative complications included higher ASA classification, specific comorbidities, and previous medical conditions. The findings align with the study’s objective of identifying significant factors impacting surgical outcomes, which include patient demographics, comorbidities, surgical techniques, and perioperative care practices. Notably, patients with a history of cancer exhibited a higher risk profile and experienced more complications compared to those without such a history. The *t*-test analysis highlighted significant differences from baseline values, suggesting these variables may be critical in predicting surgical outcomes. Although retrospective, these insights can inform future improvements in surgical care by emphasizing the importance of key risk variables, such as ASA scores and comorbidity burdens, during preoperative evaluations and informed consent discussions. Further research is necessary to validate these findings and enhance patient selection and risk stratification methods to reduce postoperative complications.

In Saudi Arabia, the Saudi Commission for Health Specialties (SCFHS) oversees the accreditation and management of residency training programs, including the five-year general surgery (GS) training program established across various hospitals in 2002. The program has seen continuous improvements aimed at enhancing the educational experience for residents. However, there is a lack of studies evaluating resident satisfaction with these programs, and existing research indicates generally low satisfaction levels among surgical residents in Saudi Arabia. In contrast, general surgery residents in the United States report significantly higher satisfaction rates, reaching up to 85.2%. The reasons behind the lower satisfaction in Saudi programs remain unclear, but identifying these factors is essential to develop strategies to improve training experiences, potentially matching the high satisfaction observed in other countries [14].

This multicenter retrospective study of 14,636 patients undergoing major general surgery in Saudi Arabia provides valuable insights into 30-day mortality and complication rates. The analysis identified several risk factors, including advanced age, higher ASA physical status, greater comorbidity burdens, longer operative times, and procedures performed during weekends or overnight hours, which were independently associated with adverse outcomes. The study underscores the need for specialized postoperative monitoring and tailored recovery protocols for high-risk patients. Implementing risk-adjustment measures and quality benchmarking based on these predictors can guide healthcare systems and providers. Despite its retrospective nature, the large sample size and diverse settings enhance the study's validity and generalizability. However, unmeasured confounding remains a concern. Prospective studies evaluating customized perioperative care bundles tailored to preoperative risk profiles are essential for improving patient outcomes. This research supports the need for personalized surgical strategies and emphasizes the importance of addressing key issues to advance healthcare quality in Saudi Arabia. Further investigations can continue to refine value-based healthcare delivery and surgical care.

The perioperative mortality rate of 0.74% observed in this study aligns with findings from similar research, such as the study at Tibebe Ghion Specialized Hospital, which also reported factors influencing postoperative mortality. This rate suggests that the surgical practices and perioperative care provided within the participating centers are generally effective. However, considering that perioperative mortality is influenced by factors such as surgical complexity, patient comorbidities, and hospital resources, there remains room for improvement. Enhancing perioperative protocols, improving patient management, and addressing any disparities in care across centers could further reduce this rate and contribute to better surgical outcomes [15].

Regarding hospitals with high ERAS compliance, there was a death within 30 days of surgery for nearly one out of every 100 patients, indicating that these procedures can be fatal if not performed properly and safely. Significant complications also affected more than 10% of patients, demonstrating the high incidence of surgery-related harm. Adverse surgical outcomes include more illnesses, longer recovery times, decreased quality of life, readmissions to the hospital, and, in extreme cases, even death. These factors have a direct impact on the health of the patient. Research has indicated that patients who experience complications after surgery tend to recover more slowly and have worse physical and mental health status one year later than patients who do not experience such problems. In addition, there is a greater chance of long-term problems such as chronic pain, reliance on medical attention, and lack of support from family members.

ERAS implementation was linked to improved outcomes. Nevertheless, compliance levels varied significantly across hospitals and protocol elements. The value of care provided to surgical patients nationwide can be further improved by standardizing care pathways and maximizing compliance.

The study's limitations include its retrospective design, which may introduce recall and selection bias, as well as unmeasured confounding variables that could affect the outcomes. The reliance on de-identified electronic medical records might lead to incomplete data and potential inaccuracies in documenting patient characteristics and procedural details. Additionally, the study's focus on a specific population in Saudi Arabia may limit the generalizability of the findings to other regions or healthcare settings. Despite these limitations, the large sample size and rigorous methodology provide valuable insights, though future prospective studies are needed to address these concerns and validate the results.

This retrospective analysis evaluated general surgery outcomes across multiple centers in Saudi Arabia. The analysis involved 14,635 patients’ electronic medical records. With regard to postoperative morbidity and mortality after major general surgery in Saudi Arabia, this study offered significant insights.

This study's findings, which assessed over 20,000 distinct surgical techniques, make up one of the most thorough retrospective evaluations of general surgical outcomes in Saudi Arabia. The findings offer several important insights with ramifications for public policy, research, and clinical practice. After 30 days, mortality rates ranging from 0.3 to 1.2% were noted; these rates were noted for all procedures and centers. It is possible that general surgical care in Saudi Arabia has advanced to a mature state, given that these rates are similar to those noted in high-volume centers worldwide. However, a higher proportion of rural centers showed higher death rates, emphasizing the need to improve district-level services [14]. Saudi Arabia's general surgery care has matured to the point where both death and morbidity rates are comparable to international benchmarks. Even though there has been progress, there is still room for improvement in terms of lowering complications by putting in place targeted, high-quality educational initiatives. Creating a standardized national surgical registry offers a strategic opportunity to advance research, health policy planning, and outcomes monitoring in the Kingdom.

This study identified key independent risk factors associated with 30-day postoperative morbidity and mortality for major general surgeries nationwide. These factors include patient characteristics, procedural details, and timing elements. The findings provide valuable insights for developing targeted enhanced recovery protocols, improving intraoperative decision-making, and refining preoperative risk counseling.

The study’s rigorous methodology, extensive sample size across multiple centers, and comprehensive clinical data enabled the identification of significant patient risk factors, procedural aspects, and healthcare delivery elements impacting postoperative outcomes. The results advocate for national efforts to optimize tailored perioperative care and underscore the importance of adjusting for biases in retrospective analyses to guide quality improvement initiatives effectively.

## CONCLUSION

This comprehensive analysis of surgical outcomes from 14,635 patients among five major hospitals in Saudi Arabia identifies key factors influencing postoperative complications and mortality. Advanced age, higher ASA classification, significant comorbidities, prolonged operative times, and procedures performed during weekends or overnight were found to be independent predictors of adverse outcomes. The study underscores the need for enhanced preoperative risk assessment and tailored postoperative care, particularly for high-risk patients. The findings highlight the importance of institutional resources and training in improving surgical outcomes. While the study provides valuable insights, its retrospective nature and focus on a specific population necessitate caution in generalizing the results. Additionally, this research can enhance preoperative risk assessment, improve postoperative care, and guide better surgical practices, ultimately aiming to improve patient outcomes and safety. Future prospective research is essential to validate these findings and develop targeted strategies to optimize surgical care and outcomes.
